# Digital droplet PCR versus quantitative PCR for lipoprotein (a) kringle IV type 2 repeat polymorphism genetic characterization

**DOI:** 10.1002/jcla.24998

**Published:** 2024-03-05

**Authors:** Giulia Barbieri, Giulia Cassioli, Ada Kura, Rebecca Orsi, Alberto Magi, Martina Berteotti, Giusi Maria Scaturro, Elena Lotti, Anna Maria Gori, Rossella Marcucci, Betti Giusti, Elena Sticchi

**Affiliations:** ^1^ Department of Experimental and Clinical Medicine University of Florence Florence Italy; ^2^ Department of Information Engineering University of Florence Florence Italy; ^3^ Atherothrombotic Diseases Center Careggi University Hospital Florence Italy; ^4^ Metabolic Diseases Unit A. Meyer Children's Hospital, University of Florence Florence Italy

**Keywords:** atherothrombotic risk, digital droplet PCR, kringle IV type 2, lipoprotein (a), quantitative real‐time PCR

## Abstract

**Background:**

Lipoprotein(a) [Lp(a)] level variability, related to atherothrombotic risk increase, is mainly attributed to *LPA* gene, encoding apolipoprotein(a), with kringle IV type 2 (KIV2) copy number variation (CNV) acting as the primary genetic determinant. Genetic characterization of Lp(a) is in continuous growth; nevertheless, the peculiar structural characteristics of this variant constitute a significant challenge to the development of effective detection methods. The aim of the study was to compare quantitative real‐time PCR (qPCR) and digital droplet PCR (ddPCR) in the evaluation of KIV2 repeat polymorphism.

**Methods:**

We analysed 100 subjects tested for cardiovascular risk in which Lp(a) plasma levels were assessed.

**Results:**

Correlation analysis between CNV values obtained with the two methods was slightly significant (*R* = 0.413, *p* = 0.00002), because of the wider data dispersion in qPCR compared with ddPCR. Internal controls C1, C2 and C3 measurements throughout different experimental sessions revealed the superior stability of ddPCR, which was supported by a reduced intra/inter‐assay coefficient of variation determined in this method compared to qPCR. A significant inverse correlation between Lp(a) levels and CNV values was confirmed for both techniques, but it was higher when evaluated by ddPCR than qPCR (*R* = −0.393, *p* = 0.000053 vs *R* = −0.220, *p* = 0.028, respectively). When dividing subjects into two groups according to 500 mg/L Lp(a) cut‐off value, a significantly lower number of KIV2 repeats emerged among subjects with greater Lp(a) levels, with stronger evidence in ddPCR than in qPCR (*p* = 0.000013 and *p* = 0.001, respectively).

**Conclusions:**

Data obtained support a better performance of ddPCR in the evaluation of KIV2 repeat polymorphism.

## Graphical abstract

In this study, real‐time and digital droplet PCR were compared in the evaluation of KIV2 repeat copy number variation polymorphism, concerning *LPA* gene, which encode apolipoprotein (a), protein component of lipoprotein (a), which is a known genetic independent risk factor for atherothrombosis. Digital droplet PCR emerges as a promising method that can make improvements in the measurement of this kind of large repeat length CNV, which is used to assess the causality between Lp(a) and cardiovascular diseases.

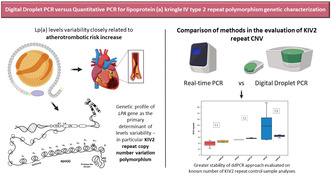

## 1 INTRODUCTION

Lipoprotein (a) [Lp(a)] is a plasma lipoprotein composed of a low‐density lipoprotein (LDL)‐particle, containing apoB‐100, which is linked by a disulphide bond to a large highly polymorphic glycoprotein named apolipoprotein (a) [apo(a)].^1^, ^2^, ^3^, ^4^ Apo(a) is characterized by the presence of loop‐like structures, called kringles (K),^5^, ^6^ that are also present in other molecules such as plasminogen, prothrombin, urokinase and tissue‐type plasminogen activator.^7^, ^8^, ^9^, ^10^ These characteristics account for Lp(a) involvement in atherosclerotic and pro‐thrombotic process, supporting its association with the pathogenesis of cardiovascular disease (CVD).^11^, ^12^, ^13^, ^14^, ^15^, ^16^ Lp(a) concentration varies from less than 1 mg/L to more than 3000 mg/L, with a skewed distribution in most populations.^17^, ^18^, ^19^ These levels are not significantly influenced by age, sex and lifestyle,^20^ and several data^21^, ^22^, ^23^ have demonstrated the genetic contribution of the *LPA* gene, encoding apolipoprotein(a), in Lp(a) levels modulation. Although genome‐wide association studies (GWAS) identified more than 200 single nucleotide polymorphisms (SNPs), which might have a possible effect on final Lp(a) concentration,^24^ a copy number variation (CNV) consisting in a variable number of a 5.6 kb repeat, including both exons 4 and 5 encoding kringle IV type 2 (KIV2) protein domain, represents its main genetic determinant, explaining around 70% of the variance in the Caucasian population.^23^, ^25^ A variable number of copies per allele, ranging from 3 to more than 40, has been described, thus generating >40 apo(a) isoforms that have different final dimensions and, in particular, small isoforms with a low number of KIV2 repeats are associated with higher plasma concentrations of Lp(a).^19^

Numerous studies confirmed the link between elevated Lp(a) plasma levels and an increase in the risk of CVD, highlighting the need for Lp(a) measurement to support patient risk stratification.^14^, ^15^, ^26^, ^27^ Nonetheless, the vast majority of available assays for measuring Lp(a) plasma levels employ antibodies directed against the repetitive motif of apo(a) protein, which is the unique component of Lp(a): due to the high degree of apo(a) size heterogeneity, this causes measurement bias, making the development of accurate methods for measuring Lp(a) plasma levels extremely challenging.^28^, ^29^

To deal with this issue, since >90% of the variance in Lp(a) levels is genetically determined, with KIV2 repeat polymorphism being the major determinant, several groups attempted to demonstrate that the characterization of this CNV can be used to indirectly assess Lp(a) levels and to evaluate its involvement in modulating cardiovascular risk.^12^, ^13^ In support of this, Kamstrup and colleagues in 2009^26^ even showed how genotyping could potentially be superior in risk prediction compared to a single plasma measurement which could be affected by passing endogenous or exogenous factors.

The gold standard methods for the evaluation of KIV2 repeat polymorphism include Western blot analysis, the most informative one since it analyses the polymorphism at the protein level, after separating the different apo(a) isoforms by SDS PAGE electrophoretic gel; Pulsed Field Gel Electrophoresis (PFGE), which provides the number of KIV2 repeats at the DNA level for each of the two apo(a) isoforms.^20^ These are both really accurate techniques but long and methodologically complex, therefore hardly applicable in the routinary diagnostic activities.

Accordingly, a faster, high‐throughput method such as quantitative real‐time PCR (qPCR) has emerged, which provides the sum of the KIV2 repeats of the two alleles, without being informative concerning genotype.^20^, ^30^ Although this method is ideal for large‐scale studies, it still has limitations in terms of stability and sensitivity.

An emerging method in a wide range of applications, including the analysis of CNVs, is digital droplet PCR (ddPCR). ddPCR is rapidly gaining in popularity over standard real‐time PCR assays for its relative ease of use, precise and accurate results, and ability to quantify nucleic acid concentration without the need for standard samples.^31^, ^32^

The number of publications in which this technology is employed for the investigation of CNVs, which can impact from single genes to regions of larger size, such as chromosomal rearrangements, continues to grow,^33^, ^34^, ^35^, ^36^, ^37^, ^38^ progressively opening up new avenues for the use of ddPCR in diagnostics.

Based on this evidence, in this preliminary study, we compare the potential of two techniques, quantitative real‐time and digital droplet PCR, for the evaluation of KIV2 repeat polymorphism, hoping that this will pave the way for discovering a rapid, cost‐effective and accurate method which could be exploited for research and diagnostic purposes in turn to improve the CVD risk classification including this genetic information.

## 2 MATERIALS AND METHODS

### 2.1 Study population

KIV2 repeat polymorphism was analysed in a population of one hundred subjects (42 males, 58 females) screened for lipid profile (total cholesterol, LDL and HDL cholesterol levels, triglycerides and Lp(a) levels), who had referred to the Metabolic Diseases Unit, Meyer Children's Hospital or to the Atherothrombotic Diseases Center, Careggi Hospital, Florence (Italy) from 2016 to 2021 for clinical evaluation of the cardiovascular risk.

Lipid parameters were obtained by routinely performed laboratory analyses. Lp(a) was measured by an immunonephelometric method (LPAX IMMAGE; Beckman Coulter, Brea, California, United States). HDL and LDL cholesterol and triglyceride levels were determined by enzymatic colorimetric assays (Roche Diagnostics GmbH, Mannheim, Germany).

### 2.2 DNA extraction

Genomic DNA was extracted from peripheral venous blood using FlexiGene Kit (Qiagen, Germany). Quantitative and qualitative assessment of genomic DNA was performed using the NanoDrop™ 1000 instrument (Thermo Fisher Scientific, MA, USA).

### 2.3 Kringle IV Type 2 (KIV2) repeats evaluation

The *LPA* KIV2 size polymorphism was genotyped using two different techniques: quantitative real‐time polymerase chain reaction, using the 7900HT Sequence Detection System (Life Technologies); digital droplet polymerase chain reaction, using the QX200 Droplet Generator and reader system (Bio‐Rad).

For both cases, genotyping resulted in an estimate of the total number (sum of repeats on both alleles) of KIV2 repeats. To improve precision, all samples were analysed in duplicates by the same molecular biologist, using the same calibrator and control samples.

Telomerase reverse transcriptase gene (*TERT*) was used as a single‐copy reference gene for both methods; primers and probes for *TERT* were commercially available and well‐validated (REF. 4,401,633, Applied Biosystems™, Waltham, MA, USA). Specific primers and probes targeting the exon 4 of the *LPA* gene were designed and generated for both techniques (Applied Biosystems™, Waltham, MA, USA); Table 1 lists the sequences for primers and probes.

**TABLE 1:** Primers and fluorogenic probe targeting exon 4 of *LPA* gene used in both quantitative and digital droplet PCR assays.

*LPA* exon 4	
Forward primer	5′‐GTC AGG TGG GAG TAC TGC AA‐3′
Reverse primer	5′‐CGA CGG CAG TCC CTT CTG‐3′
Probe	FAM‐CCT GAC GCA ATG CTC A‐MGBNFQ

### 2.4 qPCR: analysis and data normalization

qPCR was performed according to a modified protocol of previously developed assays.^12^, ^26^, ^30^ As it provides a relative quantification of samples, three internal controls – C1, C2 and C3, derived from previous analyses in which calibrator and control samples were kindly supplied by Dr. Pia R. Kamstrup^12^ – were necessary to calculate CNV values of each sample and to make them comparable within and between different plates: C2 control sample, used as reference sample, was predicted to have an intermediate number of copies (estimated repeat number: *N* = 50). As concerns C1 and C3 controls, they were predicted to have lower and higher number of repeats than C2, respectively (reported range values: 41 < C1 < 47; 61 < C3 < 68).

### 2.5 ddPCR: analysis and data normalization

ddPCR was performed using QX200™ Droplet Digital PCR (Bio‐Rad Laboratories) according to manufacturer's instructions.

Accordingly, we prepared 22 μL of a reaction mixture containing 10 ng of genomic DNA, 11 μL of 2X ddPCR Supermix for probes (no dUTP) (Bio‐Rad, Cat#1863023), 900 and 400 nM of *LPA* exon 4 primers and probe, respectively, and 1.1 μL of 20X TERT reference assay.

A 20 μL volume of this mixture was then loaded into the sample wells of a DG8 cartridge (Bio‐Rad, Cat#17005222), while 70 μL of Droplet Generator Oil (Bio‐Rad, Cat#1863005) was loaded into the oil wells. A total of 40 μL of oil–water emulsion containing approximately 12,000–20,000 droplets were generated with the QX200 droplet generator and gently transferred into a separate well of a 96‐well PCR plate. PCR was performed under the following thermocycling conditions: enzyme activation at 95°C for 10 min (1 cycle), denaturation at 94°C for 30 s and annealing/extension at 60°C for 1 min (40 cycles), enzyme deactivation at 98°C for 10 min (1 cycle) and a hold at 4°C. All steps had a ramp rate of 2°C/s. Annealing at 60°C was selected after strong gradient tests and was proven optimal (data not shown). After PCR amplification, positive and negative droplets were counted on the QX200 Droplet Reader using QuantaSoft software (v1.3.2) with automated clustering analysis. Copy number values were calculated automatically by the software based on Poisson modelling.^36^ The same control samples used for qPCR were included in each run as internal controls and in order to make data comparable between different plates.

### 2.6 Statistical analysis

Statistical analysis was performed using SPSS package v19 (SPSS Inc; Chicago, IL, USA). Categorical variables were expressed as frequencies and percentages and continuous data as median (IQR, interquartile range). To assess the difference between two independent groups, the Mann–Whitney test was used for nonparametric data. Spearman's test (two‐tailed) was used for correlation analysis. Values of *p* < 0.05 are considered statistically significant.

A Lp(a) concentration of 500 mg/L was used as a cut‐off value to compare groups.

## 3 RESULTS

Demographic and laboratory/clinical characteristics of the study population are reported in Table 2.

**TABLE 2:** Demographic and laboratory/clinical characteristics of the study population.

Characteristics	Study population *N* = 100
Age, years^a^	41.50 (14.25–61)
Male/Female	42/58
Lp(a) levels, mg/L^a^	272.50 (88.25–961.50)
Total cholesterol levels, mg/dL^a^	283 (230.75–325.50)
LDL cholesterol levels, mg/dL^a^	200.50 (153.75–247.75)
HDL cholesterol levels, mg/dL^a^	55.80 (46.50–68.00)
Triglyceride levels, mg/dL^a^	102 (73.50–162.25)

Abbreviations: HDL, high‐density lipoprotein; LDL, low‐density lipoprotein; Lp(a), lipoprotein(a).

^a^ Median (interquartile range).

**FIGURE 1:**
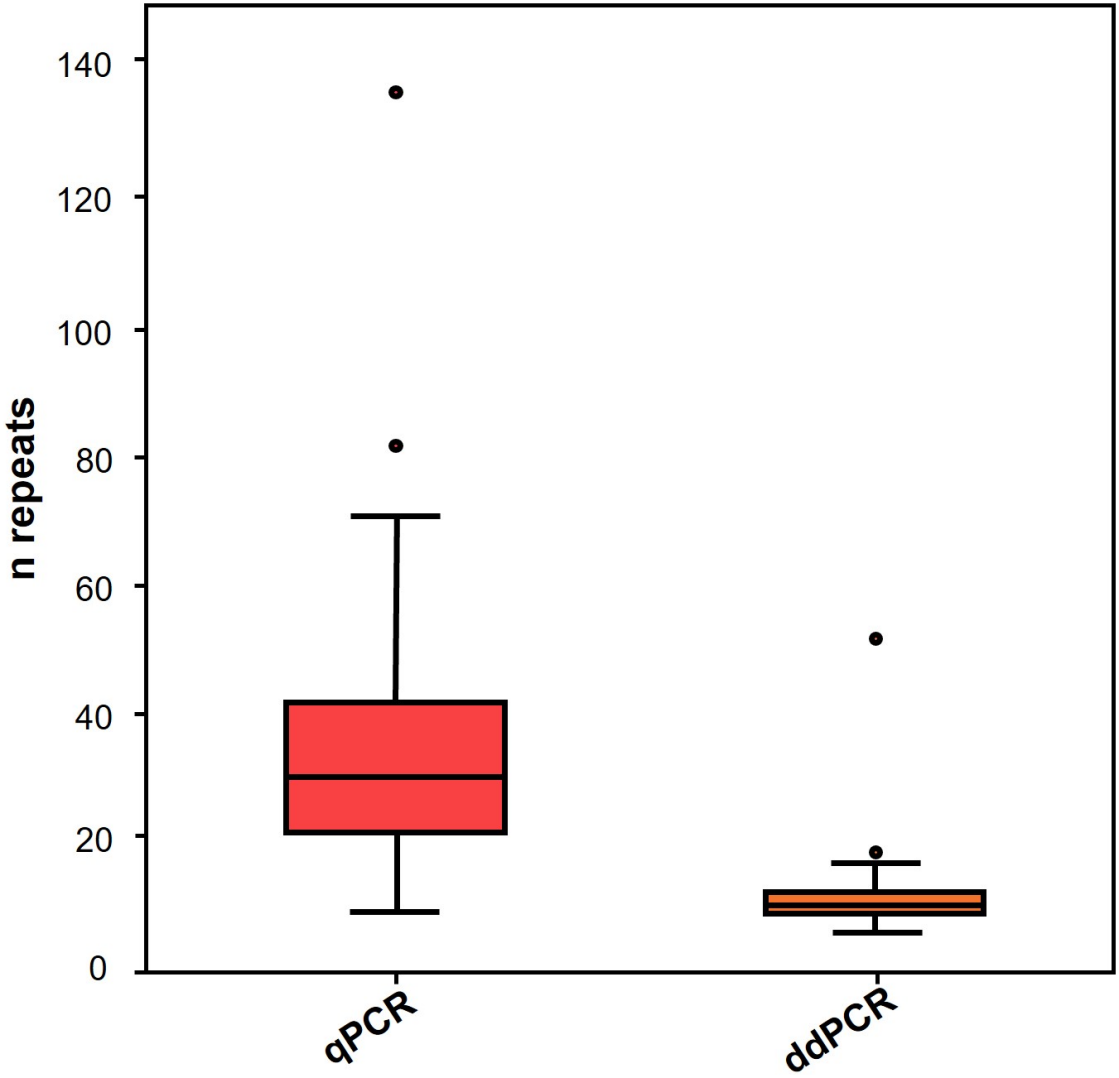
Distribution of KIV2 repeats of subjects genotyped with qPCR (on the left) and ddPCR (on the right). qPCR analysis showed a median KIV2 copy number value of 29.45 [IQR:(20.89–41.49)], while ddPCR of 10.24 [IQR:(8.92–12.26)]. ddPCR, digital droplet PCR; IQR, interquartile range; KIV2, kringle IV type 2; *n* repeats, number of repeats; qPCR, quantitative PCR.

Real‐time PCR analysis showed a median KIV2 copy number value of 29.45 [IQR:(20.89–41.49)], while median value obtained through digital droplet PCR was lower 10.24 [IQR:(8.92–12.26)]. Figure 1 shows the distribution of KIV2 repeat values assessed with the two methods within the whole cohort of subjects.

**FIGURE 2:**
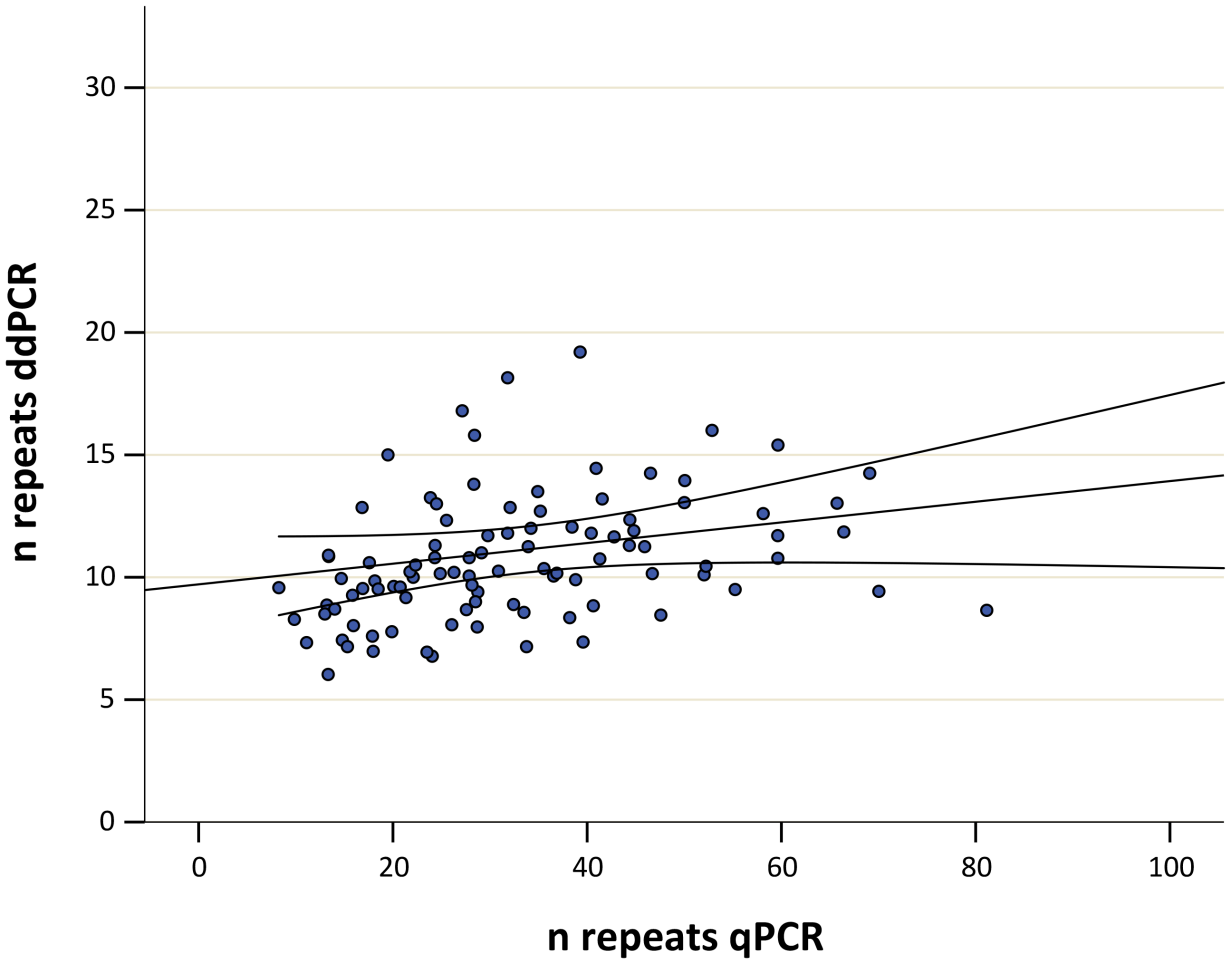
Spearman's correlation analysis between KIV2 repeats genotyped by ddPCR and qPCR (*R* = 0.413, *p* = 0.00002). ddPCR, digital droplet PCR; *n* repeats, number of repeats; qPCR, quantitative PCR.

Correlation analysis of data obtained with real‐time PCR and digital droplet PCR showed a value of *R* = 0.413, *p* = 0.00002 (Figure 2).

**FIGURE 3:**
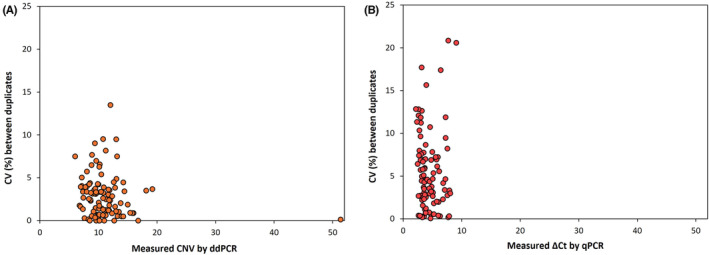
Comparison of the CV% between duplicates measured by ddPCR (A) and qPCR (B). (A) Each plot represents the CV% between CNV values obtained by duplicate samples (ranges from 0% to 13.50%). (B) Each plot represents the CV% between ΔCt values of duplicate samples (ranges from 0.04% to 20.86%). CNV, copy number variation; CV%, coefficient of variation percentage; ddPCR, digital droplet PCR; qPCR, quantitative PCR.

In order to evaluate the reproducibility of ddPCR and qPCR analysis, the intra‐assay coefficient of variation percentage (CV%) was calculated for all samples since they were run in duplicates. The CV% of ddPCR, analysed on CNV values obtained by duplicates, ranged from 0% to 13.50% (Figure 3A), while the CV% of real‐time PCR, analysed instead on ΔCt values obtained by duplicates – since CNV values are obtained in single after normalization of ΔCt values – ranged from 0.04% to 20.86% (Figure 3B).

**FIGURE 4:**
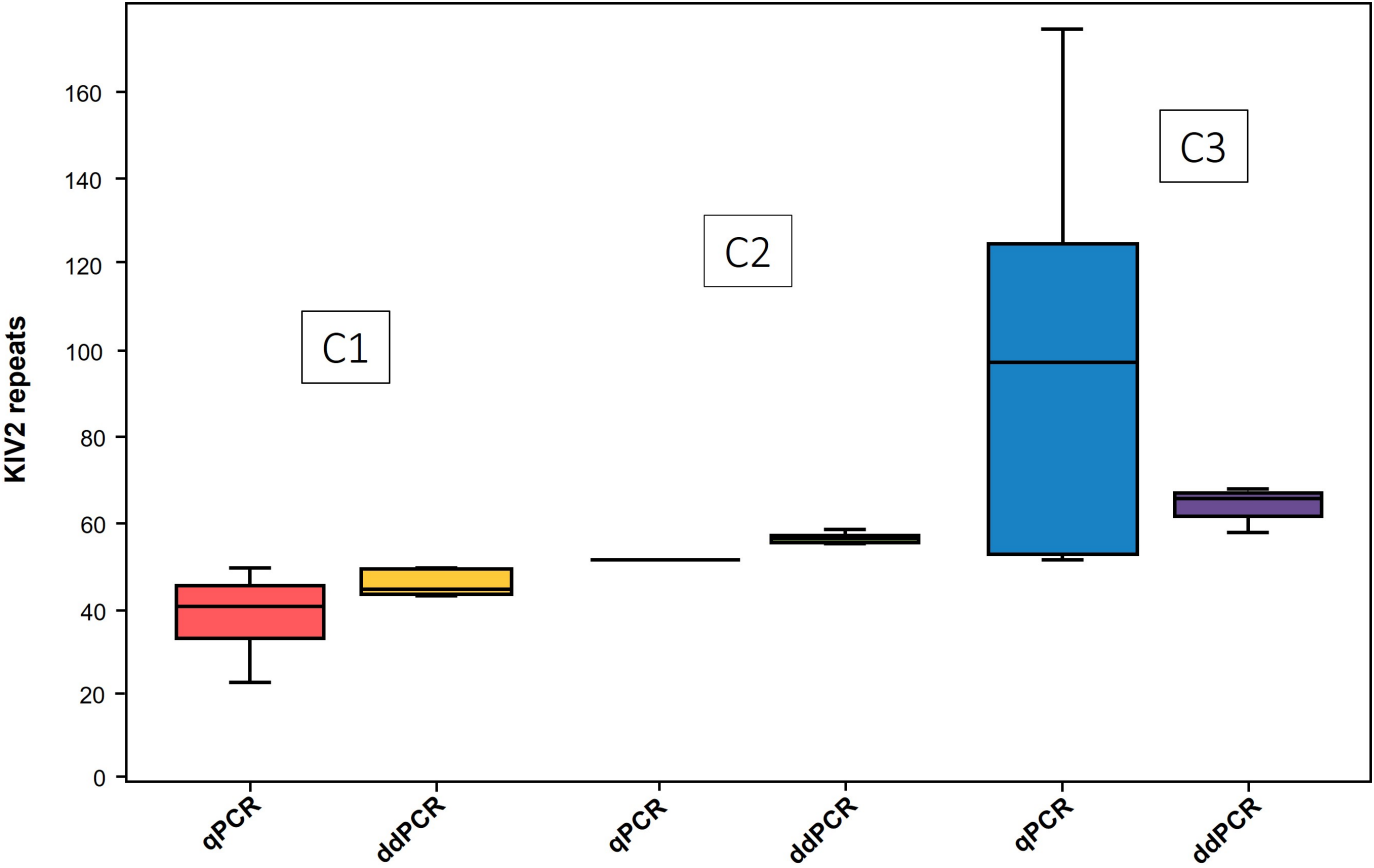
Distributions of values obtained from different experimental sessions of the C1, C2 and C3 control samples used in all plates, in both qPCR and ddPCR (C2 = 54.85 ± 1.11 with ddPCR; C1 = 37.59 ± 9.05 with qPCR vs 44.28 ± 2.74 with ddPCR and C3 = 101.18 ± 45.12 with qPCR vs 65.02 ± 3.53 with ddPCR). ddPCR, digital droplet PCR; KIV2, kringle IV type 2; qPCR, quantitative PCR.

Control sample C2, used as a reference sample for qPCR and estimated to have an intermediate number of repeats of the KIV2 domain (estimated repeats value: *N* = 50), was confirmed to have a mean value of 54.85 ± 1.11 with ddPCR analysis. Measurement of the other two internal controls C1 and C2 (estimated repeats value 41 < C1 < 47; 61 < C3 < 68) used in all the experimental sessions showed a wider data dispersion with qPCR compared to ddPCR approach [37.59 ± 9.05 vs 44.28 ± 2.74 and 101.18 ± 45.12 vs 65.02 ± 3.53, respectively] (Figure 4).

Based on these data, inter‐assay coefficient of variation percentage was calculated for both techniques to assess the reproducibility in different independent runs under repeatable conditions. CV% was calculated on CNV values for ddPCR and on ΔCt values for qPCR of each internal control in the different experimental sessions, and it was found to be much higher in qPCR than in ddPCR (Table 3).

**TABLE 3:** Comparisons between the inter‐assay CV% calculated on C1, C2 and C3 standard controls measured by ddPCR and qPCR.

Sample	MEAN ddPCR	SD ddPCR	CV% ddPCR	MEAN qPCR	SD qPCR	CV% qPCR
C1	44.28	2.74	6.19	4.59	1.43	31.22
C2	54.85	1.11	2.02	5.05	1.41	27.93
C3	65.02	3.53	5.42	5.95	1.92	32.28

Abbreviations: CV%, Coefficient of Variation Percentage; ddPCR, digital droplet PCR; qPCR, quantitative PCR; SD, Standard Deviation.

**FIGURE 5:**
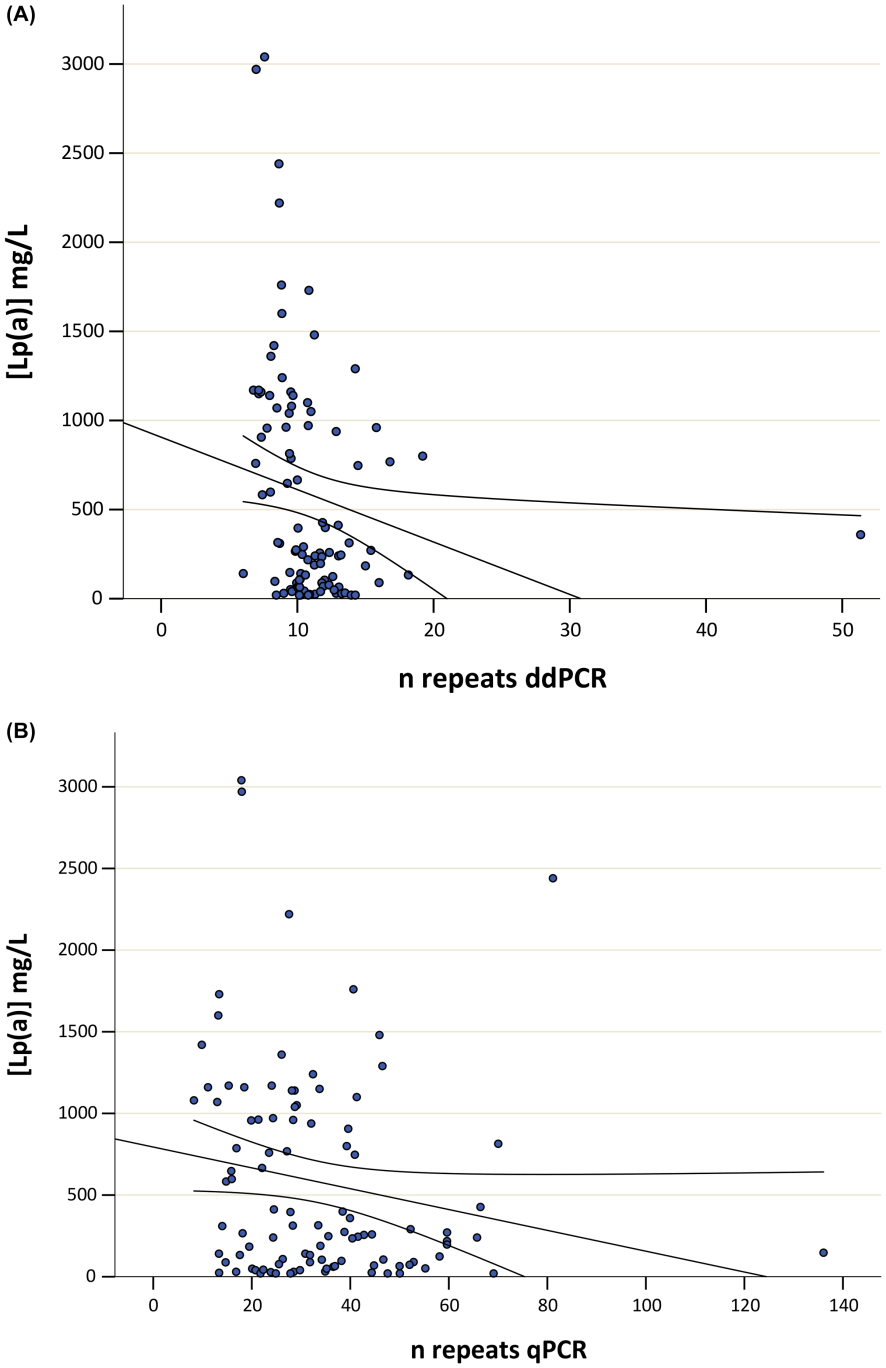
Correlation analysis between Lp(a) plasma levels of each subject and number of repeat values obtained by ddPCR (A) and by qPCR (B) – *R* = −0.393, *p* = 0.000053 vs *R* = −0.220, *p* = 0.028, respectively. Lp(a), lipoprotein(a); *n* repeats, number of repeats; ddPCR, digital droplet PCR; qPCR, quantitative PCR.

Spearman's rho test showed an inversely proportional correlation between Lp(a) levels of each subject and the copy number variation polymorphism, as expected, but higher and significant when evaluated with digital droplet PCR (Figure 5A) compared to real‐time PCR (Figure 5B). In fact, the correlation coefficient was *R* = −0.393, *p* = 0.000053 vs *R* = −0.220, *p* = 0.028, respectively.

Moreover, when dividing the cohort in two groups based on a Lp(a) cut‐off concentration of 500 mg/L, a significantly lower KIV2 repeats number emerged, with both methods, among subjects with greater levels of Lp(a), and ddPCR is better able to discriminate between the two groups than qPCR (Table 4).

**TABLE 4:** Distributions of repeats obtained with the two approaches according to the lower or higher Lp(a) concentration.

	Subjects with Lp(a) levels lower than 500 mg/L (*N* = 52)	Subjects with Lp(a) levels higher than 500 mg/L (*N* = 48)	Significance (U test Mann–Whitney)
*N* repeats ddPCR^a^	11.28 (10.06–12.81)	9.03 (7.82–10.79)	0.000013
*N* repeats qPCR^a^	35.05 (24.58–47.37)	25.19 (16.17–33.42)	0.001

Abbreviations: ddPCR, digital droplet PCR; Lp(a), lipoprotein(a); *n* repeats, number of repeats; qPCR, quantitative PCR.

^a^ Values are expressed as median (interquartile range).

## 4 DISCUSSION AND CONCLUSIONS

This study examined ddPCR and qPCR results in the evaluation of *LPA* KIV2 repeat polymorphism: the analysis of data provides support to the evidence of a greater stability and accuracy of ddPCR with respect to qPCR approach. These preliminary evaluations open up new perspectives on finding the most suitable method for Lp(a) evaluation as a cardiovascular risk factor: Lp(a) measurement has been shown to provide clinically significant improved cardiovascular risk reclassification, and therefore should be considered in subjects who have an estimated 10‐year risk of CVD that is close to the threshold between high and moderate^27^, ^39^, ^40^; nevertheless, its complex and highly variable structure makes it hard to find an accurate method to measure its concentration in plasma or serum samples. A lot of studies were able to support causality between Lp(a) and venous thromboembolism^12^ or coronary artery disease^26^ through the analysis of KIV2 repeat polymorphism and the main technique used so far was real‐time PCR. However, in many works,^37^, ^41^ the poor stability of this method is highlighted, given the need of high amount of replicates to obtain precise CNV estimates and, moreover, since it provides a relative quantification, the need of standard samples, on which data are normalized for each plate, makes it prone to errors, with loss in sensitivity.

An innovative technique that has been gaining ground over standard qPCR in a wide variety of applications is digital droplet PCR. Reaction setting up is partially similar between the two methods, and reagent costs are comparable, as well. Anyway, the peculiar workflow of ddPCR makes necessary the use of specific instrumentation and consumables, which makes it currently quite expensive.

The number of studies in which this method is used for the determination of copy number variation is increasing,^32^, ^34^, ^35^, ^36^, ^37^, ^41^, ^42^ since copy number values are calculated in an absolute way directly by the software, without the need of standard samples for normalization. A work of Sallustio et al. of 2021^37^ compared the performance of qPCR vs QuantStudio 3D digital PCR approach in determining 1q21 gain and 13q deletion frequencies: data obtained by the two methods were positively correlated, but qPCR was found to be less stable and accurate than QuantStudio digital PCR, given the need for more replicates and since going to change the calibrator sample, CNV of a same sample strongly differed.

Data obtained from the present study are in keeping with those observed by Sallustio et al.,^37^ as a higher stability in KIV2 repeats measurement is achieved with ddPCR in comparison to that observed with qPCR technique. Analysis of our data showed a significant positive correlation of CNVs obtained by the two methods, albeit not high, which probably was the result of the greater dispersion of data obtained by qPCR compared with ddPCR. In fact, we found a very huge discrepancy in results using qPCR; really, C1, C2 and C3 internal control measurement throughout different experimental sessions reported lower data dispersion and greater stability in ddPCR than in qPCR. The greater reproducibility of ddPCR was also confirmed when evaluating intra and inter‐assay coefficient of variation percentage values: intra‐assay CV% values were lower than 21% for both methods, but higher percentage values were achieved in qPCR than in ddPCR. Inter‐assay CV% first showed that both methods are more stable in the determination of intermediate CNV values and that reproducibility decreases at the extremes; however, CV% values are extremely higher when using qPCR than ddPCR.

Lp(a) levels, evaluated with an immunonephelometric assay and expressed in mass units, and KIV2 repeats number, assessed with both methods, showed a significant inverse correlation that is stronger for ddPCR than qPCR approach. Evaluating Lp(a) levels as molar concentrations (nmol/L), as previously reported,^42^, ^43^, ^44^ would have been advisable as it provides a measurement independent of the molecular weight of Lp(a) particles. Further evaluation considering Lp(a) levels in molar units might improve correlation parameters between Lp(a) concentration and CNV value of KIV2 repeat polymorphism.

Moreover, we divided subjects into two groups according to Lp(a) levels lower or higher than 500 mg/L, which represents a defined cut‐off value for increased cardiovascular risk: although lower median CNV values were observed for both methods in subjects with higher Lp(a) levels, a higher significance level was observed for ddPCR. Actually, ddPCR was more capable of discriminating between subjects with higher and lower Lp(a) levels, thus suggesting a greater accuracy of this approach and its potential in stratifying subjects' risk on the basis of KIV2 repeats number.

It has been mentioned before how ddPCR approach turns out to have a higher cost per sample compared to qPCR. In view of the present data, it could be interesting to notice, however, that ddPCR is actually a cost‐effective technique, given the added value of a greater accuracy and stability, combined with a faster execution (thus no longer requiring duplicated analyses) leading to a reduction of the cost per man‐hour. Moreover, no data normalization is required, so results are immediately available for clinical evaluation.

This is the first time in which ddPCR is used for the detection of KIV2 repeat polymorphism, known to be characterized by considerable difficulties in its determination due to its complexity related to repetitive unit length as well as extremely high variability in repeat number in the general population. Data obtained suggest the possible improvement in the measurement of this kind of large repeat length CNV by ddPCR, especially for its improved stability, which represents a great potential for a possible integration of this method in the evaluation of Lp(a) levels for clinical practice. A limitation of our study in the absolute quantification of the *LPA* KIV2 repeat numbers is the lack of the analysis of the 100 samples by a gold standard method – i.e. Western blotting or PFGE – to define accuracy of both qPCR and ddPCR.

Based on aforementioned observations, further confirmations in a larger cohort might be useful in the effort of identifying a more suitable and less time‐consuming method for the evaluation of a complex polymorphic variant, representing the main genetic determinant of Lp(a) levels. The achievement of this goal might pave the way to improve the genetic characterization of Lp(a) trait to better frame the cardiovascular risk profile of subjects.

## FUNDING INFORMATION

This work was supported by Department of Experimental and Clinical Medicine, University of Florence institutional funding.

## CONFLICT OF INTEREST STATEMENT

The authors declare that there is no conflict of interest regarding the publication of this article.

## Data Availability

All data supporting our findings are included in the article.
